# Plantar pressures are higher in cases with diabetic foot ulcers compared to controls despite a longer stance phase duration

**DOI:** 10.1186/s12902-016-0131-9

**Published:** 2016-09-15

**Authors:** Malindu E. Fernando, Robert G. Crowther, Peter A. Lazzarini, Kunwarjit S. Sangla, Scott Wearing, Petra Buttner, Jonathan Golledge

**Affiliations:** 1 Vascular Biology Unit, Queensland Research Centre for Peripheral Vascular Disease, College of Medicine and Dentistry, James Cook University, Townsville, Qld 4814 Australia; 2 Sport and Exercise, School of Health and Wellbeing, University of Southern Queensland, Ipswich, QLD Australia; 3 Department of Diabetes and Endocrinology, The Townsville Hospital, Townsville, QLD Australia; 4 Allied Health Research Collaborative, Metro North Hospital & Health Service, Queensland Health, Brisbane, Australia; 5 School of Clinical Sciences, Queensland University of Technology, Brisbane, Australia; 6 Department of Vascular and Endovascular Surgery, The Townsville Hospital, Townsville, QLD Australia; 7 Podiatry Service, Townsville Community Health Service , Townsville, QLD Australia; 8 Movement Analysis Laboratory, Sport and Exercise Science, James Cook University, Townsville, Australia; 9 Institute of Health and Biomedical Innovation, Queensland University of Technology, Brisbane, QLD Australia; 10 Centre for Chronic Disease Prevention, James Cook University, Cairns, QLD Australia; 11 Tropical Health Solutions Pty Ltd., Townsville, QLD Australia

**Keywords:** Diabetic foot disease, Biomechanics, Plantar ulcers, Peripheral diabetic neuropathy, Foot ulceration, Plantar pressure, Offloading

## Abstract

**Background:**

Current international guidelines advocate achieving at least a 30 % reduction in maximum plantar pressure to reduce the risk of foot ulcers in people with diabetes. However, whether plantar pressures differ in cases with foot ulcers to controls without ulcers is not clear. The aim of this study was to assess if plantar pressures were higher in patients with active plantar diabetic foot ulcers (cases) compared to patients with diabetes without a foot ulcer history (diabetes controls) and people without diabetes or a foot ulcer history (healthy controls).

**Methods:**

Twenty-one cases with diabetic foot ulcers, 69 diabetes controls and 56 healthy controls were recruited for this case-control study. Plantar pressures at ten sites on both feet and stance phase duration were measured using a pre-established protocol. Primary outcomes were mean peak plantar pressure, pressure-time integral and stance phase duration. Non-parametric analyses were used with Holm’s correction to correct for multiple testing. Binary logistic regression models were used to adjust outcomes for age, sex and body mass index. Median differences with 95 % confidence intervals and Cohen’s d values (standardised mean difference) were reported for all significant outcomes.

**Results:**

The majority of ulcers were located on the plantar surface of the hallux and toes. When adjusted for age, sex and body mass index, the mean peak plantar pressure and pressure-time integral of toes and the mid-foot were significantly higher in cases compared to diabetes and healthy controls (*p* < *0.05*). The stance phase duration was also significantly higher in cases compared to both control groups *(p < 0.05)*. The main limitations of the study were the small number of cases studied and the inability to adjust analyses for multiple factors.

**Conclusions:**

This study shows that plantar pressures are higher in cases with active diabetic foot ulcers despite having a longer stance phase duration which would be expected to lower plantar pressure. Whether plantar pressure changes can predict ulcer healing should be the focus of future research. These results highlight the importance of offloading feet during active ulceration in addition to before ulceration.

**Electronic supplementary material:**

The online version of this article (doi:10.1186/s12902-016-0131-9) contains supplementary material, which is available to authorized users.

## Background

A large number of studies have suggested that plantar pressures are high in people with diabetic peripheral neuropathy (DPN) and in people with a history of diabetic foot ulcers (DFUs) [1–12]. It has been proposed that high plantar pressure predispose people with DPN to develop DFUs [13, 14]. Hence current international guidelines advocate achieving at least a 30 % reduction in maximum plantar pressure to reduce the risk of developing DFUs [13]. While it is accepted that high plantar pressures in people with DPN lead to DFUs and remain high following DFUs, it is not known if plantar pressures are elevated at the time of active DFUs [10]. To complicate the matter, the few studies which have investigated barefoot plantar pressure in people with active DFUs have major inconsistencies in the populations studied and reported results [6, 8, 12, 14–16]. Several studies have investigated heterogeneous cohorts of people either with a history of DFUs or with an active DFU [6, 12, 16], whilst another study only investigated male patients [14]. In addition, some studies have reported plantar pressures in a limited number of sites [8, 15] or alternatively reported aggregated plantar pressure from multiple sites [11, 16]. These inconsistent approaches make it difficult to interpret whether plantar pressures are actually elevated in people with active plantar DFUs [10, ﻿^17^–^19^].

In a recent meta-analysis of observational studies, we reported that plantar pressures were not significantly different in people with active DFUs compared to controls with DPN without active DFUs [10]. The result from this analysis may have been due to a lack of statistical power to detect a true difference, or due to the fact that plantar pressures are not significantly different in cases with active DFUs compared to controls [10]. This prospective study investigated a homogenous cohort of people with active DFUs and assessed a number of plantar pressure measures recorded at multiple sites on the plantar surface of both feet. We hypothesised that cases with active DFUs would have higher magnitudes and durations of plantar pressure compared to controls [8, 10]. Therefore, the aim of this study was to assess whether plantar pressures were higher in patients with active unilateral plantar DFUs of >3 months duration (cases) compared to patients without a foot ulcer history (diabetes controls) and patients without a diabetes or foot ulcer history (healthy controls).

## Methods

### Study design and criteria for inclusion

The full protocol for this study is published elsewhere [20]. Cases with type-2 diabetes mellitus and DFUs (DFU group), type-2 diabetes controls (DMC group) and healthy controls (HC group) were recruited for this case-control study [20]. Exclusion criteria for all groups included: (1) orthopaedic, musculoskeletal, vestibular, visual or neurological problems affecting mobility (other than DPN); (2) previous orthopaedic surgical intervention of the lower limb; (3) diabetes types other than type-2 diabetes; (4) peripheral arterial disease defined as an ankle-brachial pressure index (ABPI) of < 0.8 in either limb or a past history of or diagnosis or treatment of peripheral arterial disease; (5) planned vascular reconstructions in the subsequent 12 months; and (6) pregnancy [20]. The exclusion criteria were designed to avoid inclusion of people with problems impacting on mobility that would likely mask the impact of a plantar ulcer on gait.

The DFU and DMC groups were recruited from inpatient wards, outpatient clinics and community health clinics within the Townsville Hospital and Health Service District, Queensland, Australia [20]. The HC group were recruited through community advertising and from staff at the university where the study took place. All participants were recruited in the period July 2012 to May 2014. The study was approved by two human research ethics committees [20].

### Sample size calculation and case-control matching

Utilising previous research in patients with DPN without foot ulcers [21], we estimated that 28, 112 and 56 participants were required in the DFU, the DMC and the HC groups, respectively. This was determined using a one-way analysis of variance (ANOVA) with 80 % power, an overall significance of 0.05 adjusted for multiple tests (maximum of 8) to detect a 20 % difference in forefoot plantar pressure, and a ratio of 1 DFU case: 4 DMCs: 2 HCs. We attempted to match the sex and age (to within 5 years) of controls and cases. We selected an age range of 5 years to make recruitment feasible and as we deemed this would be sufficient to reduce any impact that differences in the ages of cases and controls may have on the outcomes.

### Anthropometric and clinical assessments

All anthropometric and clinical measurements were performed according to previously published protocols [20]. The same assessor (MEF) carried out all assessments. Briefly, height was assessed using a wall mounted telescopic metal stadiometer (Seca model 220, Seca Scales, Hamburg, Germany). Body weight was measured using bioelectrical impedance scales (TANITA TBF 521, TANITA Corporation, Illinois, United States of America). Body Mass Index (BMI) was calculated by dividing the participant’s body mass (kg) by the square of the participant’s height (m). A standardised metal measuring tape (KDS F10-02, Muratech-KDS Corporation, Osaka, Japan) was used to assess hip and waist circumference while the participant stood in a relaxed positon with feet together and arms freely hanging to the side. Good-to-excellent reproducibility (concordance correlation coefficients between 0.999 [95 % Confidence Interval (CI): (0.999-0.999)] and 0.998 [95 % CI: (0.995–0.999)]) have been reported for all measurements [20].

Foot structure and the presence of orthopaedic foot abnormalities were assessed in all participants by a trained podiatrist (MEF). Lesser toe deformities, foot abnormalities and arch contours of the feet along with presence and grade of hallux abducto valgus (HAV) deformity were assessed [22], utilising a set protocol and recognised standards of assessment as described previously [20]. An extensive account of the methodology used to assess ABPIs, the monofilament score, Michigan Neuropathy Symptom Score (MNSI), Physical Assessment Score and haematological markers, specifically glycated haemoglobin A1C) (HbA1C) and estimated glomerular filtration rate (eGFR) is reported in our published protocol [20].

### Assessment of plantar pressure and stance phase duration

A Footscan® pressure plate (RSScan International, Olen, Belgium) was used for plantar pressure assessment. This plate was 2 m in length, 0.4 m in width and contained 16384 sensors, with individual sensor dimensions of 0.0076 m x 0.0051 m. All pressure data were captured at a rate of 100 Hz. The three step approach to capturing data was chosen [23]. This method has been previously validated and involves each participant taking two steps before landing on the pressure plate [23]. A standard protocol for collecting dynamic barefoot plantar pressure data was established prior to commencing data collection [19, 20]. We did not use a walkway during gait and did not adjust stepping preference. Instead stepping preference was controlled by asking participants to start walking with the dominant foot throughout and by commencing from the same distance away from the platform. Participants were given time to familiarise themselves with the protocol. Briefly, this protocol included using five gait assessments per participant as reported in previous literature [24].

**Fig. 1:**
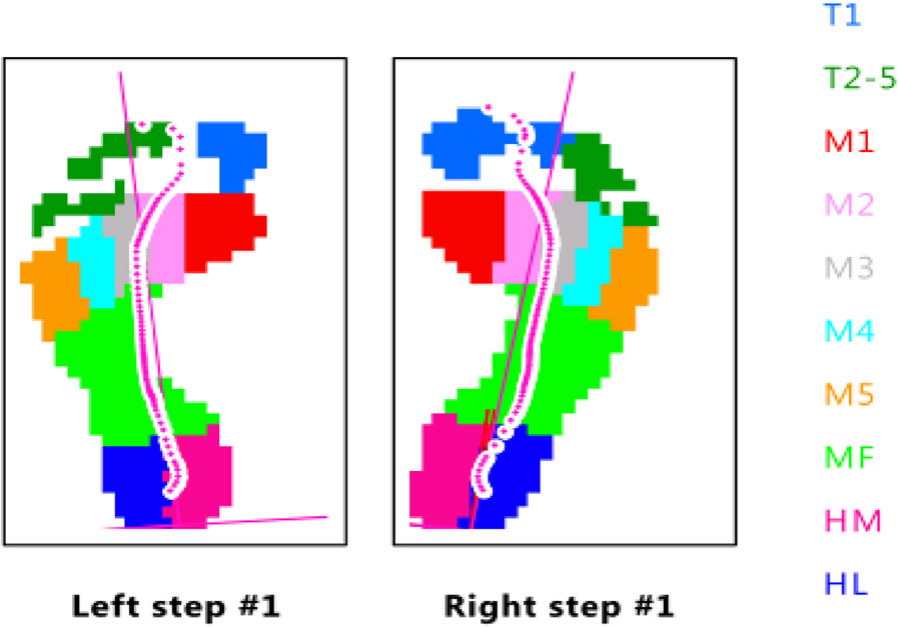
Example of allocation of masks to plantar sites. Legend: T1 = toe 1, T2-5 = toes 2 to 5, M1 = metatarsal 1, M2 = metatarsal 2, M3 = metatarsal 3, M4 = metatarsal 4, M5 = metatarsal 5, MF = mid-foot, HM = medial heel, HL = lateral heel

Footscan® processing software (version 8.01) was used to mask each electronic footprint into ten sites. The locations included the plantar surfaces of the hallux, toes 2–5, metatarsal one, metatarsal two, metatarsal three, metatarsal four, metatarsal five, the mid-foot, the lateral rear-foot and the medial rear-foot (see Fig. 1) [19]. Current debate exists as to which plantar pressure measure is most appropriate for investigating people at risk of DFUs [24]. Therefore we measured a range of different plantar pressure outcomes including: mean peak pressure (mpp) in N/cm^2^ which quantifies the magnitude of pressure; pressure-time integral (pti) in Ns/cm^2^ which quantifies the duration and magnitude of pressure; maximum sensor pressure (msp) in N/cm^2^ and the contact area in cm^2^. The stance-phase duration (length of time in milliseconds that the feet were in contact with the platform) during plantar pressure assessment was recorded and averaged for the five selected trials for each participant as a surrogate measure of gait speed [25].

The mpp and pti and stance phase duration were considered as primary outcome measures. The maximum sensor pressure and contact area were considered as secondary outcomes. These measurements were reported by the software for the left and right foot for each participant during barefoot gait [20]. We have previously reported the reproducibility of plantar pressure assessments [19]. Mpp and pti measurements assessed at most anatomical locations typically resulted in coefficients of variations (cvs) below 30 % and were more reproducible than msp measurements [19].

### Statistical analysis

SPSS 20.0 for Windows (SPSS Inc., Illinois, United States of America) was used for statistical analyses. Non-parametric analyses were selected as the majority of continuous data were not normally distributed. A *p*-value of <0.05 was used throughout as an indicator of statistical significance. Descriptive statistics were reported by groups for continuous and categorical variables and consisted of sample size, median and interquartile range [IQR] or numbers and percentages (%). These were statistically compared between the three patient groups initially, followed by *post hoc* (between two-group) tests. Diabetes related outcomes (HbA1c, diabetes duration, overall monofilament score, MSNI scores, eGFR and insulin use) were only compared between the DFU and DMC groups. We used the Kruskal-Wallis test and the Mann-Whitney *U* test or the Pearson’s chi-square tests. We only used the Fishers exact test, if expected frequencies were less than or equal to five for categorical outcomes.

To test our main hypothesis, the plantar pressure data for the ulcerated foot in cases were compared to the average plantar pressures from the left and right foot in the appropriate control group. Initial comparisons of the primary outcomes (mpp, pti and stance phase duration) and secondary outcomes (contact area and msp) were performed using the Kruskal-Wallis test, followed by *post hoc* comparisons using the Mann-Whitney *U* test. We also carried out paired analyses between the relevant descriptive factors and outcome measures of the ulcerated and non-ulcerated feet of the DFU group using the Wilcoxon Signed Rank and McNemar’s tests. As multiple outcomes were tested in this study, we corrected the *p*-values from primary and secondary outcome test results using the Holm step-wise correction [26]. Stance phase duration was not corrected. Between two-group comparisons were reported as estimated differences in the median (Δ) with 95 % CIs using Hodges-Lehmann estimates from ranks [27].

Binary logistic regression analyses were used to adjust all significant post-hoc comparison outcomes for age, sex and BMI. Odds Ratios (ORs) and 95 % CIs of binary logistic regression results were computed; significant differences were flagged and reported within Additional file 1. Only outcome data which was significant after correction and adjustment were reported in the results section of the paper and within the main data Tables. All other data (including secondary outcome data) were reported in Additional file 1. In addition to estimated differences in the median, Cohen’s d (standardised mean difference) was calculated for all variables which remained significant after adjustment and correction, to assess their effect size using a revised formula for skewed data: Cohen’s *d* = *median 1-median 2/pooled IQR* [28]. Effect-size magnitudes were used to estimate the degree of difference [29]. The size of the difference was graded based on Cohen’s d as: <0.10 trivial difference; 0.10–0.20 small difference; 0.20–0.60 medium difference; 0.60–1.20 large difference and ≥1.20 a very large difference [29].

## Results

### Participant recruitment and statistical power

From 208 participants that were screened for the study, 146 were recruited. This included 21 in the DFU group, 69 in the DMC group and 56 in the HC group. Due to difficulty in recruitment, a 1:3:3 matching process replaced the originally planned 1:4:2. A post-hoc power test on this recruited sample suggested a power > 80 % remained using the assumptions of the other sample size calculation.

### Demographic, clinical and foot characteristics

The demographic and clinical characteristics of the participants are displayed in Table 1. The DMC group was older than the HC group *(p < 0.05)*. The two diabetes groups had more males, a greater weight and BMI compared to the HC group *(all p < 0.05)*. The DFU group also had a longer diabetes duration, more insulin use, lower eGFR and higher MNSI neuropathy scores than the DMC group (*all p < 0.05*). The foot morphological characteristics are reported in Table 2. No significant difference in foot characteristics existed between the three groups, except hammertoe deformity which was more commonly present in the DFU group compared to the DMC and HC groups (*p < 0.01*). There were also no significant difference in foot characteristics when comparing ulcerated and non-ulcerated feet within the DFU group (see Table 2). The plantar ulcer locations of the DFU group were at the lateral heel (*n* = 2), mid-foot (*n* = 3), medial forefoot (*n* = 2), central forefoot (*n* = 1), lateral forefoot (*n* = 1), apex of lesser digits (*n* = 5), and hallux (*n* = 7).

**Table 1:** Clinical and demographical characteristics of the study cohort by group

	DFU group (*n* = 21)	DMC group (*n* = 69)	HC group (*n* = 56)	*P* value
Age (years)	66.0 [52.0–72.0]	63.0 [58.0–72.0]^b^	56.0 [55.0–73.0]	*P = 0.005*
Males [number and %]	15 (71.4 %)^b^	46 (66.7 %)^b^	24 (42.9 %)	*P = 0.011*
Ethnicity [number and %]				
Caucasian	20 (95.2 %)	65 (94.2 %)	54 (96.4 %)	*P = 0.660*
Australian Aboriginal/Indigenous/Torres-strait Islander	1 (4.8 %)	2 (2.9 %)	2 (3.6 %)	
Other	-	2 (2.9 %)	-	
Diabetes duration [years]	17.00 [14.5–20.5]	7.5 [4.0–16.5]	-	*P = 0.008*
Height (cm)	175.1 [164.8–179.0]	170.0 [163.0–177.5]	170.0 [164.0–174.3]	*P = 0.199*
Weight (kg)	99.6 [82.3–125.1]^b^	92.3 [80.1–100.7]^b^	72.9 [64.1–81.6]	*P < 0.001*
BMI (Body Mass Index)	32.3 [27.0–37.9]^b^	31.0 [29.0–33.4]^b^	25.8 [23.0–29.3]	*P < 0.001*
Waist to Hip Ratio	1.0 [0.9–1.1]	0.9 [0.9–1.0]	0.9 [0.9–0.9]	*P = 0.099*
Hba1c (mmol/l)	55.5 [46.7–66.5]	51.0 [44.0–61.0]	-	*P = 0.514*
Uses Insulin [number and %]	13 (61.9 %)^a^	19 (27.5 %)	-	*P < 0.001*
Smoking Status [number and %]				
Never Smoked	14 (66.7 %)	34 (49.3 %)	26 (46.4 %)	*P = 0.201*
Ex-Smoker	6 (28.6 %)	29 (42.0 %)	29 (51.8 %)	
Current Smoker	1 (4.8 %)	6 (8.7 %)	1 (1.8 %)	
Overall lowest ABPI	1.1 [0.9–1.2]	1.1 [1.0–1.2]	1.0 [1.0–1.1]	*P = 0.838*
Overall Monofilament Score (out of 20)	5.00 [2.5–14.0]^﻿a^	20.00 [17.5–20.0]	20.00 [20.0–20.0]	*P < 0.001*
MNSI Symptom Score (DPN)	7.00 [6.0–8.0]^a^	5.00 [3.0–6.0]	-	*P < 0.001*
MNSI Physical Assessment Score (DPN)	6.00 [6.0–8.0]^a^	2.00 [1.0–3.0]	-	*P < 0.001*
eGFR	71.0 [56.2–83.5]^a^	85.0 [71.0–91.0]	-	*P = 0.044*

Data displays median and [IQR] and number and percentages (%). All analyses performed were non-parametric. This involved Pearson’s chi-squared tests for categorical variables and Kruskal-Wallis test for comparisons between three groups and Man-Whitney *U* test for between DFU and DMC group comparisons and for post-hoc testing between two groups. A significance level of *p* = 0.05 was used throughout. Diabetes duration indicates fractions of years living with type-2 diabetes mellitus. ABPI = ankle brachial pressure index. ABPI values are for ulcerated limbs of the DFU group and the lowest reported in the control groups (DMC, HC). eGFR = estimated glomerular filtration rate (a marker of renal function). Monofilament score is out of a total of 20, measured at ten sites for each foot. MNSI scores indicate the total scores from the Michigan Neuropathy Screening Instrument in relation to the neuropathy symptom score and physical assessment score. ^a^ = *p* <0.05 when compared to the DMC group in post-hoc analysis ^b^=*p* < 0.05 compared to the HC group in post hoc analysis

**Table 2:** Foot characteristics of the study cohort by group

Explanatory measure	DFU group (*n* = 21)		DMC group (*n* = 69)	HC group (*n* = 56)	*P value*	*P value for ulcerated vs. non-ulcerated feet of cases [paired]*
	Ulcerated feet	Non-ulcerated feet^b^				
Pes planus feet type	14 (66.7 %)	12 (60.0 %)	29 (42.0 %)	19 (33.9 %)		
Normal arched feet type	4 (19.0 %)	4 (20.0 %)	23 (33.3 %)	20 (35.7 %)	*0.146*	*0.317*
Pes cavus feet type	3 (14.3 %)	4 (20.0 %)	17 (24.6 %)	17 (30.4 %)		
First MTPJ rom (degrees)	30.0 [25.0–45.0]	33.5 [27.5–45.0]^a,b^	44.0 [30.0–50.0]	45.0 [35.0–60.0]	*0.077*	*0.404*
Ankle Joint rom (restricted dorsiflexion)	17 (81.0 %)	16 (80.0 %)	51 (73.9 %)	33 (58.9 %)	*0.281*	*0.368*
Subtalar Joint rom (restricted inversion/eversion)	2 (9.5 %)	1 (5.0 %)	3 (4.4 %)	2 (3.6 %)	*0.885*	*0.846*
Hallux Abducto Valgus deformity^*^						
(No deformity)	14 (66.7 %)	14 (70.0 %)	51 (73.9 %)	30 (53.6 %)	*0.132*	*0.392*
(Grade 1)	5 (23.8 %)	4 (20.0 %)	13 (18.8 %)	15 (26.8 %)		
(Grade 2)	1 (4.8 %)	1 (5.0 %)	3 (4.3 %)	10 (17.9 %)		
(Grade 3)	1 (4.8 %)	1 (5.0 %)	2 (2.9 %)	1 (1.8 %)		
Claw toe deformity	6 (28.6 %)	8 (40.0 %)	11 (15.9 %)	15 (26.8 %)	*0.252*	*0.500*
Hammer toe deformity	12 (57.1 %)^a,b^	10 (50.0 %)^a,b^	16 (23.2 %)	9 (16.1 %)	*0.001*	*0.625*
Mallet toe deformity	3 (14.3 %)	5 (25.0 %)	14 (20.3 %)	8 (14.3 %)	*0.630*	*0.500*

McNemar’s test was performed to assess paired significances between the ulcerated and non-ulcerated feet of the DFU group for categorical outcome and the Wilcoxon Signed Rank test was used to assess continuous variables. ^a^=*p* <0.05 when compared to the DMC group in post-hoc analysis ^b^=*p* < 0.05 compared to the HC group in post hoc analysis. ^*^Hallux Abducto Valgus (HAV) deformity grades were based on the Manchester scale [22] as reported in the study protocol [20]. ^**^These outcomes were calculated with a denominator of 20 due to missing data for the non-ulcerated foot of one participant in the case group. *rom*, range of motion

### Primary outcomes

All primary outcome data are reported in Table 3 and in more detail in Additional file 1. The DFU group had significantly higher mpp and pti of the toes 2–5 and mid-foot sites compared to the DMC and HC groups (*all p < 0.05*). The mpp of metatarsal 1 was also significantly higher in the DFU group compared to the HC group (*p < 0.05*). The DFU also had significantly longer stance phase duration compared to the DMC and HC groups (*p < 0.05*).

**Table 3:** Plantar pressure characteristics of the primary outcome measures by group

Outcome measure	DFU group (*n* = 21)	DMC group (*n* = 69)	HC group (*n* = 56)	*Corrected P-value*	Median difference DFU *vs.* DMC [95 % CI of difference]	Median difference DFU *vs.* HC [95 % CI of difference]	Cohen’s D DFU *vs.* DMC	Cohen’s D DFU *vs.* HC
Mean Peak plantar Pressure (mpp) N/cm^2^								
Toes 2–5	3.0 [2.4–5.6]^﻿a,b﻿^	2.5 [1.9–3.1]	2.1 [1.8–2.7]	*P = 0.007*	-0.8 [-1.5–(-)0.9]	-1.0 [-1.9–(-)0.5]	0.21	0.40
Metatarsal 1	5.7 [4.5–8.5]^b^	5.5 [4.3–6.4]	4.6 [3.8–5.4]	*P = 0.008*	-	-1.5 [-2.7–(-)0.5]	-	0.36
Mid-foot	3.8 [3.1–6.5]^a,b^	3.0 [2.5–3.7]	2.2 [1.8–2.9]	*P < 0.001*	-0.9 [-2.0–(-)0.3]	-1.7 [-3.0–(-)1.1]	0.32	0.63
Pressure-time integral (pti) Ns/cm^2^								
Toes 2–5	0.9 [0.6–1.4]^a,b^	0.6 [0.5–0.8]	0.5 [0.3–0.6]	*P = 0.001*	0.3 [0.1–0.6]	-0.4 [-0.8–(-)0.2]	0.45	0.60
Mid-foot	1.7 [1.2–2.5]^a,b^	1.0 [0.8–1.3]	0.6 [0.4–0.9]	*P = 0.001*	0.6 [0.3–1.0]	-1.0 [-1.3–(-)-0.7]	0.67	1.07
Stance phase duration (ms)								
	820 [752–960]^a,b^	739 [699–788]	703 [669–748]	P < 0.001*	-84 [-140–(-)34]	-115 [-163–(-)66]	0.51	0.75

Data displays median and [IQR] values based on the ulcerated feet of the DFU group compared to the reported maximum values in the DMC and HC groups. Corrected *p*-values indicate *p*-values obtained after Holm correction rounded to 3 decimal places. Average stance phase duration indicates the average time the left and right feet were in contact with the ground in milliseconds (ms) from heel contact to toe-off. *Stance phase duration *p*-values were not corrected as this was analysed as an independent variable. All above reported plantar pressure outcome data were significant on post-hoc two-way tests and remained significant after adjusting for age, sex and BMI. Binary logistic regression analyses were only performed for variables which were significant on the post-hoc test. Cohen’s d was only calculated for variables which were significantly different after adjustment. (-) = not computed as this was not significantly different. See Additional file 1 for odds ratios and additional data. ^a^=*p* < 0.05 when compared to the DMC group in post-hoc analysis ^b^=*p* < 0.05 compared to the HC group in post hoc analysis

### Secondary outcomes

All secondary outcome results and paired analyses results are reported in Additional file 1: Tables S2, S3, S4, S5, S6 and S7. The DFU group had higher msp of toes 2–5 and higher contact areas of the mid-foot and metatarsal 1 compared to both the DMC group and the HC group *(p < 0.05).* The DFU group had a higher msp of the mid-foot compared to the DMC group (*p < 0.05*). None of the plantar pressure outcomes were statistically significant between the ulcerated and non-ulcerated feet after correction in paired analyses (see Additional file 1: Tables S6 and S7).

## Discussion

To the best of our knowledge this is the first prospective study to have simultaneously examined such an extensive assessment of plantar pressures in a homogenous group of people with active DFUs [20]. Our primary results show the mpp (representing magnitudes of plantar pressure) and the pti (representing the duration and magnitude of plantar pressure) at toes 2–5 and the mid-foot were significantly higher in cases with DFUs compared to both control groups. Eight out of twenty-one ulcers in cases occurred at these plantar sites. Secondary results show the msp at the toes and the contact area of the mid-foot were also significantly higher in cases with DFUs compared to both controls. These findings occurred despite a longer stance phase duration representing slower gait speed in cases with DFUs. Consistent with our hypothesis, although cases with DFUs walked slower, their forefoot plantar pressures (especially at toes 2–5) were significantly higher compared to controls without DFUs.

The plantar pressure data in our diabetes control group were similar to that of a previous study that used the same plantar pressure platform [30]. We chose not to control gait speed in our participants as it has been reported that people with diabetes walk more cautiously than healthy controls [31]. However, previous studies have identified that gait speed can significantly alter the distribution and magnitude of plantar pressures [32, 33]. Faster gait speeds (i.e. shorter stance phase durations) have been shown to increase plantar pressure at the heel, medial and central forefoot and the toes 2–5 while decreasing plantar pressure beneath the mid-foot and lateral forefoot [32, 33]. This has been termed a medialisation of the loading pattern [33]. Conversely, a slower gait speed, as denoted by the longer stance phase duration, would be expected to produce higher plantar pressures beneath the mid-foot and lateral forefoot but lower plantar pressures at all other sites.

While our finding of elevated mid-foot pti and mpp in cases is consistent with a slower walking speed, our finding that mpp was elevated beneath toes 2–5 is counter to this effect. Therefore, despite a longer stance phase, higher plantar pressures still occur in cases with active DFUs. In a previous meta-analysis we proposed a ‘guarded gait strategy’ may be used by people with active DFUs to reduce plantar pressure acting on the ulcerated foot [10]. Our current findings however are contrary to the presence of a ‘guarded gait strategy’ which would have resulted in lower plantar pressure in our cases. Our findings suggest that a longer stance phase is inadequate to lower the plantar pressure beneath the ulcerated foot during gait [34]. Many factors such as severity of DPN, foot deformity, excess weight and altered gait patterns have all been implicated as potential causes of elevated plantar pressure in people with DFUs [14, 31].

A study by Stokes et al. (1975) suggested a mechanical aetiology to DFUs in people with DPN [6]. They reported that plantar DFUs may occur at sites of maximal load in people with DPN [6]. They also reported that there was a lateral shift of the maximum pressure on the forefoot and a decrease in the plantar pressure of the toes in people with DPN [6]. Conversely, our study demonstrated higher plantar pressures beneath toes 2–5 in cases with active DFUs. This finding may be due to several reasons including the use of more modern equipment with greater sensitivity and greater spatial resolution in our study, or the differences in the populations studied. Stokes et al. had a heterogeneous cohort with only two participants with active DFUs while all our twenty-one cases had active plantar DFUs.

Several DFUs in our cases were located in the toe region. A significantly higher proportion of participants in the DFU group also had a hammer-toe deformity of the lesser toes. A recent study by Barn and colleagues investigated predictors of barefoot plantar pressure in people with DPN with a history of DFUs [35]. Barn and colleagues found that the presence of local deformity (such as toes and foot deformities) were the largest contributing factors to raised barefoot dynamic plantar pressure in their population [35]. The presence of hammer-toe deformity was the largest single contributor towards elevated plantar pressure at the lesser toes [35], consistent with other research [14, 36]. It is possible that the higher plantar pressures seen in the cases in our study may have been associated with the presence of hammer-toe deformity as it has been previously associated with an increased risk of ulceration due to mechanical load placed on toes during gait [37]. Interestingly, no differences in plantar pressures were observed between the ulcerated and non-ulcerated feet in paired analyses and this may have been due the high number of bilateral toe deformities in our cases. In our study, the severity of Hallux Abducto Valgus deformity was not different between groups which align with Barn and colleagues findings that the presence of Hallux Abducto Valgus was not a predictor of plantar pressure [35]. Additionally, all other foot characteristics showed no differences between groups or within cases, suggesting that unlike hammer-toe deformities, other foot characteristics may have a lesser effect on plantar pressure [14, 17, 38].

The pti is defined as the area under the peak–pressure–time curve and has been used to study ulceration because it incorporates pressure as well as time, both of which are suggested to be important in DFU formation [15, 39]. In agreement with the view that reporting the pti in addition to the mpp may be counterproductive [9, 24, 40], the results from our study demonstrate that both the pti and the mpp were significantly higher at toes and at the mid-foot in cases with DFUs compared to either control group. Conversely, the effect-sizes of the differences in pti measurements were much larger compared to those of mpp. This observation is consistent with a recent study in people with DPN without DFUs that identified pti was significantly higher in five out of ten possible regional comparisons, as opposed to the mpp, which was significantly higher only in three out of ten comparisons [41].

Another study reported that the difference in pti between the metatarsal heads and the hallux was far greater in people with DFUs compared to controls without DFUs [15]. Bacarin et al. have also previously found that people with DPN and a history of DFUs have a significantly higher pti at the mid-foot after controlling for gait speed [42]. Currently, what the pti represents in the context of ulceration is still uncertain [24]. A longitudinal analysis of both the pti and mpp measurements in a cohort of people with healing and non-healing DFUs may provide information regarding the importance of these two parameters on ulcer healing [15, 20]. For example, it may be possible that one parameter may be more predictive of ulcer healing. A longitudinal analysis will also provide observations regarding the variability of plantar pressures in cases with DFUs when compared to controls without DFUs over-time [23].

In contrast to the findings of the current study, Sauseng et al. found that the maximum plantar pressure and contact area was higher at plantar metatarsal 1 but was lower at metatarsal 4, metatarsal 5 and at the mid-foot in people with DFUs compared to controls [15]. The different findings in the current study may be due to a number of factors. Firstly, in the current study plantar pressures were examined in 10 locations in both feet however Sauseng et al. only studied seven locations [15]. Sauseng and co-workers pooled plantar pressure data from the ulcerated and non-ulcerated feet of cases, debrided plantar callus prior to plantar pressure evaluation, and did not report gait speed or stance phase duration. They also studied a group of patients with very few DFUs occurring at toes 2–5 as a majority of the DFUs were located on the plantar surfaces of the metatarsals in their study [15]. Nevertheless, the results from Sauseng and co-workers are in alignment with our results in indicating that ulcer location is an important predictor of the site of high plantar pressure and that higher plantar pressure may occur at ulcer sites in people with DFUs. Interestingly, Sauseng and co-workers found that the mpp at the hallux was not higher in people with DFUs compared to controls, despite the fact that some DFUs occurred at the hallux, which was also the case in our study [15]. We were unable to show any difference in the range of motion of the first metatarsophalangeal joint between groups in our study. The reason why we were unable to see higher plantar pressures at the hallux in people with DFUs may be due to the fact that they were able to limit the amount of loading on the hallux using the available range of motion at the first metatarsophalangeal joint. This is consistent with our finding of higher mpp at this site in our cases compared to healthy controls.

Plantar pressures are theoretically the result of the vertical force exerted on the foot during gait divided by the contact area. Therefore, assuming that the spatial resolution of the sensors were adequate and the plantar skin surface was completely in contact with the pressure platform during measurement [17], either vertical ground reaction force has to increase, or the total contact area for a given site has to decrease in order for plantar pressure to be elevated. We have demonstrated increased plantar pressures and larger contact areas in cases with DFUs. These results highlight that vertical ground reaction forces are also likely to be significantly elevated in cases with DFUs. This is consistent with recent findings in the same cohort [43]. To our knowledge, although it is often speculated, increased ground reaction forces have not been previously reported in people with active DFUs until recently [43]. These findings suggest that people with active DFUs experience significantly higher mechanical stresses during gait. On the other hand, while plantar pressures represent only the vertical component of the applied tissue stress, shear-forces are also a crucial consideration in the formation of DFUs [41]. As the same local area under the foot can experience stresses in opposite directions, investigation of shear forces in cases with DFUs will provide further information regarding tissue stresses [44]. The increased contact areas observed in our cases at several sites is in agreement with the idea that ground reaction forces other than the vertical force (i.e. shear forces) may also be important in DFU formation [41]. This is consistent with the finding that shear forces in the anterior–posterior direction during gait in the same cohort of people with DFUs was significantly higher than in controls [43]. Future studies should focus on assessing shear-pressures, especially at sites of active ulcers.

Our study has a number of limitations and strengths. We were unable to adjust all our analyses for multiple factors such as foot deformities, arch type and neuropathy severity due to relatively small group sizes. We examined barefoot gait rather than shod gait and purposefully did not control gait speed as we wanted to examine the natural gait characteristics of our participants. We believe that by imposing minimal constraints, the observed gait would be consistent with the participant’s everyday gait pattern. We used stance phase duration as a surrogate measure of gait speed. We were, however, unable to focus our investigation on individual ulcer sites due to a small sample-size and resultant lack of statistical power and this area still requires investigation. We believe that our findings, however, are consistent with plantar pressures representative of a majority of cases who had DFUs in the forefoot region. There are differences in plantar pressure values obtained using different platforms with different resolutions and various methods of assessment, which is a clear limitation in the field [45]. Our plantar pressure results seem to be lower than other values reported in the literature, but are consistent with others using the same platform to assess participants with diabetes [30]. The strengths of our study include the use of reproducible methodology to capture plantar pressure, reporting the reproducibility of plantar pressure acquisition prior to this study [19] and the use of a conservative statistical approach.

## Conclusions

In summary, this study has demonstrated that plantar pressures are higher in cases with active unilateral diabetic foot ulcers compared to diabetes and healthy controls without ulcers. Higher plantar pressures occurred in cases despite a longer stance phase duration which would be expected to lower plantar pressures. This highlights the importance of offloading feet during active ulceration to overcome the mechanical impact of elevated plantar pressures on ulcerated tissue. Evaluating plantar pressures throughout ulcer progression may provide further clarity on the relationship between plantar pressures and the mechanical stresses experienced by patients with active foot ulcers.

## Additional file

Additional file 1: Additional results and Supplementary Tables 1 to 8 for main results (before correction and adjustment) and after correction and adjustment and results of additional analyses. (DOCX 58 kb)

## Abbreviations

Abpi: Ankle brachial pressure index
Ca: Contact area
DFU: Diabetes foot ulcer
DPN: Diabetic Peripheral Neuropathy
Mpp: Mean peak plantar pressure
Msp: Maximum sensor pressure
PAD: Peripheral Arterial Disease
Pti: Pressure-time integral
﻿Rom: Range of motion﻿﻿﻿
Vgrf: Vertical ground reaction force

## References

[1] Veves A, Murray HJ, Young MJ, Boulton AJM (1992). The risk of foot ulceration in diabetic patients with high foot pressure: a prospective study. Diabetologia.

[2] Wrobel JS, Najafi B (2010). Diabetic foot biomechanics and gait dysfunction. J Diabetes Sci Technol.

[3] Frykberg RG, Lavery LA, Pham H, Harvey C, Harkless L, Veves A (1998). Role of neuropathy and high foot pressures in diabetic foot ulceration. Diabetes Care.

[4] Pham H, Armstrong DG, Harvey C, Harkless LB, Giurini JM, Veves A (2000). Screening techniques to identify people at high risk for diabetic foot ulceration: a prospective multicenter trial. Diabetes Care.

[5] Hokkam EN (2009). Assessment of risk factors in diabetic foot ulceration and their impact on the outcome of the disease. Prim Care Diabetes.

[6] Stokes IA, Faris IB, Hutton WC (1975). The neuropathic ulcer and loads on the foot in diabetic patients. Acta Orthop Scand.

[7] Ctercteko GC, Dhanendran M, Hutton WC, Le Quesne LP (1981). Vertical forces acting on the feet of diabetic patients with neuropathic ulceration. Br J Surg.

[8] Kanade RV, van Deursen RW, Harding K, Price P (2006). Walking performance in people with diabetic neuropathy: benefits and threats. Diabetologia.

[9] Waaijman R, Bus SA (2012). The interdependency of peak pressure and pressure-time integral in pressure studies on diabetic footwear: no need to report both parameters. Gait Posture.

[10] Fernando ME, Crowther RG, Pappas E, Lazzarini PA, Cunningham M, Sangla KS, Buttner P, Golledge J (2014). Plantar pressure in diabetic peripheral neuropathy patients with active foot ulceration, previous ulceration and no history of ulceration: a meta-analysis of observational studies. PLoS ONE.

[11] Boulton AJ, Hardisty CA, Betts RP, Franks CI, Worth RC, Ward JD, Duckworth T (1983). Dynamic foot pressure and other studies as diagnostic and management aids in diabetic neuropathy. Diabetes Care.

[12] Armstrong DG, Peters EJ, Athanasiou KA, Lavery LA (1998). Is there a critical level of plantar foot pressure to identify patients at risk for neuropathic foot ulceration?. J Foot Ankle Surg.

[13] Bus SA, Armstrong DG, Van Deursen R, Lewis J, Caravaggi C, Cavanagh PR (2016). IWGDF guidance on footwear and offloading interventions to prevent and heal foot ulcers in patients with diabetes. Diabetes Metab Res Rev.

[14] Cavanagh PR, Sims DS, Sanders LJ (1991). Body mass is a poor predictor of peak plantar pressure in diabetic men. Diabetes Care.

[15] Sauseng S, Kästenbauer T, Sokol G, Irsigler K (1999). Estimation of risk for plantar foot ulceration in diabetic patients with neuropathy. Diabetes Nutr Metab.

[16] Brash PD, Foster JE, Vennart W, Daw J, Tooke JE (1996). Magnetic resonance imaging reveals micro-haemorrhage in the feet of diabetic patients with a history of ulceration. Diabet Med.

[17] Cavanagh PR, Ulbrecht JS, Caputo GM. New Developments In The Biomechanics Of The Diabetic Foot. Diabetes Metab Res Rev. 2000;16 Suppl 1:S6-10. 10.1002/1520-7560(200009/10)16:1+<::aid-dmrr130>3.0.co;2-z 11054880

[18] Wearing SC, Urry S, Smeathers JE, Battistutta D (1999). A comparison of gait initiation and termination methods for obtaining plantar foot pressures. Gait Posture.

[19] Fernando M, Crowther R, Cunningham M, Lazzarini P, Sangla K, Buttner P, Golledge J (2016). The reproducibility of acquiring three dimensional gait and plantar pressure data using established protocols in participants with and without type 2 diabetes and foot ulcers. J Foot Ankle Res.

[20] Fernando ME, Crowther RG, Cunningham M, Lazzarini PA, Sangla KS, Golledge J (2015). Lower limb biomechanical characteristics of patients with neuropathic diabetic foot ulcers: the diabetes foot ulcer study protocol. BMC Endocr Disord.

[21] Savelberg HH, Schaper NC, Willems PJ, de Lange TL, Meijer K (2009). Redistribution of joint moments is associated with changed plantar pressure in diabetic polyneuropathy. BMC Musculoskelet Disord.

[22] Garrow AP, Papageorgiou A, Silman AJ, Thomas E, Jayson MI, Macfarlane GJ (2001). The grading of hallux valgus. The Manchester Scale. J Am Podiatr Med Assoc.

[23] Bus SA, de Lange A (2005). A comparison of the 1-step, 2-step, and 3-step protocols for obtaining barefoot plantar pressure data in the diabetic neuropathic foot. Clin Biomech.

[24] Hafer JF, Lenhoff MW, Song J, Jordan JM, Hannan MT, Hillstrom HJ. Reliability of plantar pressure platforms. Gait Posture. 2013;38:544–8. 10.1016/j.gaitpost.2013.01.028 PMC3732469 23454044

[25] Kirtley C, Whittle MW, Jefferson RJ (1985). Influence of walking speed on gait parameters. J Biomed Eng.

[26] Ludbrook J (1998). Multiple comparison procedures updated. Clin Exp Pharmacol Physiol.

[27] Hodges JL, Lehmann EL. Estimates of Location Based on Rank Tests. Ann Math Statist. 1963;34(2):598-611.

[28] Cohen JA (1988). Statistical power analysis for the behavioral sciences.

[29] Hopkins WG. A scale of magnitudes for effect statistics. A new view of statistics Sports Science.Org: Sports Science 2002 [cited 2015 29th August]. Available (http://sportsci.org/resource/stats/effectmag.html).

[30] Qiu X, Tian DH, Han CL, Chen W, Wang ZJ, Mu ZY, Liu KZ (2015). Plantar pressure changes and correlating risk factors in Chinese patients with Type 2 Diabetes: preliminary 2-year results of a prospective study. Chin Med J (Engl).

[31] Ko M, Hughes L, Lewis H (2012). Walking speed and peak plantar pressure distribution during barefoot walking in persons with diabetes. Physiother Res Int.

[32] Burnfield JM, Few CD, Mohamed OS, Perry J (2004). The influence of walking speed and footwear on plantar pressures in older adults. Clin Biomech (Bristol, Avon).

[33] Rosenbaum D, Hautmann S, Gold M, Claes L (1994). Effects of walking speed on plantar pressure patterns and hindfoot angular motion. Gait Posture.

[34] Fernando M, Crowther R, Lazzarini P, Sangla K, Cunningham M, Buttner P, Golledge J (2013). Biomechanical characteristics of peripheral diabetic neuropathy: a systematic review and meta-analysis of findings from the gait cycle, muscle activity and dynamic barefoot plantar pressure. Clin Biomech (Bristol, Avon).

[35] Barn R, Waaijman R, Nollet F, Woodburn J, Bus SA (2015). Predictors of barefoot plantar pressure during walking in patients with diabetes, peripheral neuropathy and a history of ulceration. PLoS One.

[36] Mueller MJ, Hastings M, Commean PK, Smith KE, Pilgram TK, Robertson D, Johnson J (2003). Forefoot structural predictors of plantar pressures during walking in people with diabetes and peripheral neuropathy. J Biomech.

[37] Ledoux WR, Shofer JB, Smith DG, Sullivan K, Hayes SG, Assal M, Reiber GE (2005). Relationship between foot type, foot deformity, and ulcer occurrence in the high-risk diabetic foot. J Rehabil Res Dev.

[38] Molines-Barroso RJ, Lázaro-Martínez JL, Aragón-Sánchez FJ, Álvaro-Afonso FJ, García-Morales E, García-Álvarez Y. Forefoot ulcer risk is associated with foot type in patients with diabetes and neuropathy. Diabetes Research and Clinical Practic. 2016; (In Press). 10.1016/j.diabres.2016.01.008 26810268

[39] Stess RM, Jensen SR, Mirmiran R (1997). The role of dynamic plantar pressures in diabetic foot ulcers. Diabetes Care.

[40] Keijsers NL, Stolwijk NM, Pataky TC (2010). Linear dependence of peak, mean, and pressure-time integral values in plantar pressure images. Gait Posture.

[41] Yavuz M (2014). Plantar shear stress distributions in diabetic patients with and without neuropathy. Clin Biomech (Bristol, Avon).

[42] Bacarin TA, Sacco IC, Hennig EM (2009). Plantar pressure distribution patterns during gait in diabetic neuropathy patients with a history of foot ulcers. Clinics (Sao Paulo).

[43] Fernando ME, Crowther RG, Lazzarini PA, Sangla KS, Buttner P, Golledge J (2016). Gait parameters of people with diabetes-related neuropathic plantar foot ulcers. Clin Biomech.

[44] Yavuz M, Tajaddini A, Botek G, Davis BL (2008). Temporal characteristics of plantar shear distribution: relevance to diabetic patients. J Biomech.

[45] Ledoux WR, Shofer JB, Cowley MS, Ahroni JH, Cohen V, Boyko EJ (2013). Diabetic foot ulcer incidence in relation to plantar pressure magnitude and measurement location. J Diabetes Complicat.

