# Supplementary material for: Plantar pressures are higher in cases with diabetic foot ulcers compared to controls despite a longer stance phase duration
Source: BMC Endocr Disord. 2016 Sep 15;16:51. doi: 10.1186/s12902-016-0131-9 (PMC5024422; doi:10.1186/s12902-016-0131-9)
Supplement: Additional file 1: — Additional results and Supplementary Tables 1 to 8 for main results (before correction and adjustment) and after correction and adjustment and results of additional analyses. (DOCX 58 kb) [file 12902_2016_131_MOESM1_ESM.docx]

**Additional File 1: Additional Results Tables**

**Additional Table 1: Mean peak pressure and pressure time integral characteristics of the cohort by group**

|  | DFU Group (n=21) | DMC Group (n=69) | HC Group (n=56) | p value | Corrected p-value |
| --- | --- | --- | --- | --- | --- |
| **Plantar Pressure (mpp)** **) (N/cm²)** |  |  |  |  |  |
| Toe 1/ Hallux | 3.6 [2.5-8.2] | 4.0 [3.1-5.0] | 3.5 [2.9-4.8] | 0.347 | 0.347 |
| Toes 2-5 | 3.0 [2.4-5.6] | 2.5 [1.9-3.1] | 2.1 [1.8-2.7] | 0.001 | 0.007 |
| Metatarsal 1 | 5.7 [4.5-8.5] | 5.5 [4.3-6.4] | 4.6 [3.8-5.4] | 0.002 | 0.008 |
| Metatarsal 2 | 6.8 [4.9-9.2] | 7.0 [6.0-7.9]  .0.0 | 5.7 [4.8-7.1] | 0.003 | 0.009 |
| Metatarsal 3 | 6.6 [4.7-9.9] | 7.2 [5.9-8.3] | 5.8 [4.4-7.3] | 0.004 | 0.009 |
| Metatarsal 4 | 5.7 [4.1-7.6] | 6.1 [5.2-7.1] | 4.8 [4.0-5.7] | <0.001 | <0.001 |
| Metatarsal 5 | 4.7 [3.4-6.2] | 4.9 [3.9-5.8] | 3.6 [2.8-4.7] | <0.001 | <0.001 |
| Mid Foot | 3.8 [3.1-6.5] | 3.0 [2.5-3.7] | 2.2 [1.8-2.9] | <0.001 | <0.001 |
| Medial Heel | 5.8 [4.5-9.2] | 6.1 [5.3-6.8] | 5.2 [4.4-6.0] | 0.001 | 0.007 |
| Lateral Heel | 6.3 [4.8-9.6] | 6.1 [5.5-7.1] | 5.1 [4.2-6.6] | 0.001 | 0.007 |
| **Pressure Time Integral (pti) (Ns/cm²)** |  |  |  |  |  |
| Toe 1/ Hallux | 0.9 [0.5-1.9] | 1.0 [0.7-1.2] | 0.8 [0.6-1.1] | 0.231 | 0.231 |
| Toes 2-5 | 0.9 [0.6-1.4] | 0.6 [0.5-0.8] | 0.5 [0.3-0.6] | 0.0001 | 0.001 |
| Metatarsal 1 | 2.3 [1.5-2.9] | 1.8 [1.4-2.4] | 1.4 [1.1-1.7] | 0.0001 | 0.001 |
| Metatarsal 2 | 2.7 [1.7-3.7] | 2.5 [2.0-2.9] | 1.9 [1.5-.4] | 0.0001 | 0.001 |
| Metatarsal 3 | 2.9 [1.7-3.9] | 2.6 [2.0-3.1] | 1.5 [1.6-2.7] | 0.002 | 0.004 |
| Metatarsal 4 | 2.3 [2.0-3.3] | 2.3 [2.0-2.8] | 1.7 [1.4-2.2] | 0.0001 | 0.001 |
| Metatarsal 5 | 1.9 [1.3-2.7] | 1.8 [1.4-2.1] | 1.2 [0.9-1.7] | 0.0001 | 0.001 |
| Mid Foot | 1.7 [1.2-2.5] | 1.0 [0.8-1.3] | 0.6 [0.4-0.9] | 0.0001 | 0.001 |
| Medial Heel | 2.3 [1.5-3.6] | 2.0 [1.6-2.3] | 1.5 [1.2-1.9] | 0.0001 | 0.001 |
| Lateral Heel | 2.2 [1.5-3.6] | 2.0 [1.6-2.3] | 1.5 [1.1-1.9] | 0.0001 | 0.001 |

Legend: Data displays median and [IQR] values based on the ulcerated and non-ulcerated feet of the DFU group compared to the average values in the DMC and HC groups. The Kruskal-Wallis test was used for all between group comparisons with a significance of p<0.05 and these are the main p-values reported. The corrected p-value indicates p-values after performing the Holm step-wise correction for multiple testing. A significance of p <0.05 was used throughout.

**Additional Table 2: Contact area and maximum sensor pressure characteristics of the cohort by group**

|  | DFU Group (n=21) | DMC Group (n=69) | HC Group (n=56) | p value | Corrected p-value |
| --- | --- | --- | --- | --- | --- |
| **Max Sensor Pressure (msp) (N/cm²)** |  |  |  |  |  |
| Toe 1/ Hallux | 17.9 [7.4-30.7] | 14.5 [10.0-18.5] | 13.7 [10.4-18.4] | 0.696 | 0.696 |
| Toes 2-5 | 11.7 [8.3-23.9] | 8.4 [6.8-11.3] | 8.0 [6.3-9.8] | 0.001 | 0.009 |
| Metatarsal 1 | 19.5 [10.8-27.5] | 13.2 [10.8-17.4] | 12.5 [9.5-16.3] | 0.015 | 0.120 |
| Metatarsal 2 | 18.0 [12.7-21.4] | 17.4 [14.2-21.2] | 15.7 [12.5-19.0] | 0.195 | 0.588 |
| Metatarsal 3 | 17.0 [12.3-24.3] | 16.7 [13.3-20.6] | 15.1 [12.4-18.6] | 0.165 | 0.588 |
| Metatarsal 4 | 14.1 [10.6-15.9] | 12.9 [10.7-16.6] | 12.0 [9.3-15.0] | 0.147 | 0.588 |
| Metatarsal 5 | 11.7 [8.1-15.8] | 11.7 [9.0-15.1] | 8.8 [6.9-12.8] | 0.016 | 0.120 |
| Mid Foot | 12.4 [9.4-19.4] | 9.3 [7.7-11.1] | 6.7 [5.6-8.7] | 0.0001 | 0.001 |
| Medial Heel | 17.1 [11.9-26.7] | 15.4 [13.2-18.5] | 14.4 [11.8-17.0] | 0.076 | 0.380 |
| Lateral Heel | 16.2 [11.4-28.2] | 15.9 [13.5-19.3] | 13.8 [10.8-17.4] | 0.021 | 0.126 |
| **Contact Area (ca) ( cm²)** |  |  |  |  |  |
| Toe 1/ Hallux | 11.0 [8.4-13.3] | 10.9 [9.3-12.3] | 11.3 [9.5-12.4]  3.3 | 0.694 | 0.694 |
| Toes 2-5 | 10.1 [8.0-11.7] | 8.4 [6.4-10.2] | 8.6 [6.5-10.1] | 0.044 | 0.132 |
| Metatarsal 1 | 12.8 [10.2-15.2] | 11.1 [10.0-12.2] | 10.1 [8.6-10.6] | 0.0001 | 0.001 |
| Metatarsal 2 | 10.0 [8.7-11.9] | 9.7 [8.7-10.4] | 9.0 [8.4-9.6] | 0.002 | 0.014 |
| Metatarsal 3 | 8.9 [7.5-9.7] | 8.1 [7.5-8.9] | 7.9 [7.2-8.4] | 0.018 | 0.072 |
| Metatarsal 4 | 9.5 [8.5-10.3] | 8.6 [8.0-9.5] | 8.5 [8.0-9.1] | 0.014 | 0.070 |
| Metatarsal 5 | 8.5 [6.6-10.6] | 9.4 [8.2-10.6] | 9.0 [7.8-9.7] | 0.081 | 0.165 |
| Mid Foot | 37.4 [28.1-42.4] | 26.9 [22.0-32.4] | 21.7 [13.9-25.9] | 0.0001 | 0.001 |
| Medial Heel | 17.4 [15.5-18.8] | 16.1 [14.2-17.5] | 15.1 [13.6-15.7] | 0.0001 | 0.001 |
| Lateral Heel | 14.9 [13.8-15.7] | 14.0 [12.4-15.5] | 13.5 [12.2-14.1] | 0.003 | 0.018 |

Legend: Data displays median and [IQR] values based on the ulcerated and non-ulcerated feet of the DFU group compared to the average values in the DMC and HC groups. The Kruskal-Wallis test was used for all between group comparisons with a significance of p<0.05 and these are the main p-values reported. The corrected p-value indicates p-values after performing the Holm step-wise correction for multiple testing. A significance of p <0.05 was used throughout.

**Additional Table 3: Post-hoc test results and median differences by group**

|  | **DFU Group (n=21)** | **DMC Group (n=69)** | **HC Group (n=56)** | **Corrected**  **p-value** | **Median difference DFU vs. DMC**  **[95% CI of difference]** | **Median difference DFU vs. HC**  **[95% CI of difference]** |
| --- | --- | --- | --- | --- | --- | --- |
| **Plantar Pressure (mpp)** **) (N/cm²)** | | | | | | |
| Toes 2-5 | 3.0 [3.1]^ab^ | 2.5 [1.1] | 2.1 [0.9] | 0.007 | -0.8 [-1.5-(-)0.9] | -1.0 [-1.9-(-)0.5] |
| Metatarsal 1 | 5.7 [4.0]^b^ | 5.5 [2.1] | 4.6 [1.6] | 0.008 | - | -1.5 [-2.7-(-)0.5] |
| Metatarsal 2 | 6.8 [4.3] | 7.0 [1.9]  .0.0 | 5.7 [2.3] | 0.009 | - | - |
| Metatarsal 3 | 6.6 [5.2] | 7.2 [2.5] | 5.8 [2.8] | 0.009 | - | - |
| Metatarsal 4 | 5.7 [3.5] | 6.1 [1.9 | 4.8 [1.7] | <0.001 | - | - |
| Metatarsal 5 | 4.7 [2.8]^b^ | 4.9 [1.9] | 3.6 [1.8] | <0.001 | - | -1.1 [-2.0-(-)0.1] |
| Mid Foot | 3.8 [3.4]^ab^ | 3.0 [1.1] | 2.2 [1.1] | <0.001 | -0.9 [-2.0-(-)0.3] | -1.7 [-3.0-(-)1.1] |
| Medial Heel | 5.8 [4.7]^b^ | 6.1 [1.4] | 5.2 [1.6] | 0.007 | - | -1.0 [-2.3-(-)0.8] |
| Lateral Heel | 6.3 [4.8]^b^ | 6.1 [1.6] | 5.1 [2.3] | 0.007 | - | -1.3 [-2.7-(-)0.2] |
| **Max Sensor Pressure (msp) (N/cm²)** | | | | | | |
| Toes 2-5 | 11.7 [15.7]^ab^ | 8.4 [4.5] | 8.0 [3.5] | 0.009 | 3.8 [1.1-8.6] | -4.6 [-9.5-(-)1.9] |
| Mid Foot | 12.4 [10.0]^ab^ | 9.3 [3.5] | 6.7 [3.1] | 0.001 | 3.8 [1.4-6.8] | -5.8 [-8.8-(-)3.4] |
| **Pressure Time Integral (pti) (Ns/cm²)** | | | | | | |
| Toes 2-5 | 0.9 [0.9]^ab^ | 0.6 [0.3] | 0.5 [0.3] | 0.001 | 0.3 [0.1-0.6] | -0.4 [-0.8-(-)0.2] |
| Metatarsal 1 | 2.3 [1.4]^b^ | 1.8 [0.9] | 1.4 [0.6] | 0.001 | - | -0.8 [-1.2-(-)0.1] |
| Metatarsal 2 | 2.7 [2.0]^b^ | 2.5 [10] | 1.9 [0.9] | 0.001 | - | -0.7 [-1.3-(-)0.1] |
| Metatarsal 3 | 2.9 [2.2]^b^ | 2.6 [1.0] | 1.5 [2.0] | 0.004 | - | -0.8 [-1.5-(-)0.1) |
| Metatarsal 4 | 2.3 [1.4]^b^ | 2.3 [0.8] | 1.7 [0.8] | 0.001 | - | -0.7 [-1.1-(-)0.2] |
| Metatarsal 5 | 1.9 [1.4]^b^ | 1.8 [07] | 1.2 [0.8] | 0.001 | - | -0.7 [-1.1-(-)0.3] |
| Mid Foot | 1.7 [1.4]^ab^ | 1.0 [0.5] | 0.6 [0.4] | 0.001 | 0.6 [0.3-1.0] | -1.0 [-1.3-(-)-0.7] |
| Medial Heel | 2.3 [2.1]^b^ | 2.0 [0.7] | 1.5 [0.6] | 0.001 | - | -0.8 [-1.4-(-)0.2] |
| Lateral Heel | 2.2 [2.1]^b^ | 2.0 [0.7] | 1.5 [0.8] | 0.001 | - | -0.7 [-1.4-(-)0.2] |
| **Contact Area (ca) ( cm²)** | | | | | | |
| Metatarsal 1 | 12.8 [5.0]^ab^ | 11.1 [2.3] | 10.1 [2.0] | 0.001 | -2.0 [-3.4-(-)0.4] | -3.0 [-5.0-(-)1.6] |
| Metatarsal 2 | 10.0 [3.2]^b^ | 9.7 [1.8] | 9.0 [1.3] | 0.014 | - | -1.2 [-2.4-(-)0.3] |
| Mid Foot | 37.4 [14.3]^ab^ | 26.9 [10.4] | 21.7 [12.4] | 0.001 | -8.4 [-13.5-(-)3.7] | -15.1 [20.0-(-)9.7] |
| Medial Heel | 17.4 [3.3]^b^ | 16.1 [3.3] | 15.1 [2.1] | 0.001 | - | -2.2 [-3.3-(-)1.2] |
| Lateral Heel | 14.9 [1.9]^b^ | 14.0 [3.1] | 13.5 [1.9] | 0.018 | - | -1.6 [-2.5-(-)0.7] |

Legend: Data displays median and [IQR] values based on the ulcerated and non-ulcerated feet of the DFU group compared to the reported maximum values in the DMC and HC groups. The Kruskal-Wallis test was used for all between group comparisons with a significance of p<0.05 and these are the main p-values reported. The Man Whitney U test was used for Posthoc comparisons for all significant outcomes. ^a^= p <0.05 when compared to the DMC group in post-hoc analyses ^b^ =p<0.05 compared to the HC group in post hoc analyses. - = not statistically significant on Posthoc analyses and therefore median differences were not reported. Hodges-Lehmann estimates of the median difference and 95% confidence intervals are reported.

**Additional Table 4: Post-hoc test results and odds ratios from binary logistic regression**

|  | **Median difference DFU vs. DMC**  **[95% CI of difference]** | **OR**  **[95% CI]** | **Median difference DFU vs. HC**  **[95% CI of difference]** | **OR**  **[95% CI]** |
| --- | --- | --- | --- | --- |
| **Plantar Pressure (mpp)** **) (N/cm²)** | | | | |
| Toes 2-5 | -0.8 [-1.5-(-)0.9] | 2.2 [1.3-3.8]^ | -1.0 [-1.9-(-)0.5] | 4.9 [1.3-18.0]^ |
| Metatarsal 1 | - |  | -1.5 [-2.7-(-)0.5] | 1.9 [1.1-3.4]^ |
| Metatarsal 5 | - |  | -1.1 [-2.0-(-)0.1] | 1.3 [0.9-2.0] |
| Mid Foot | -0.9 [-2.0-(-)0.3] | 2.0 [1.3-3.2]^ | -1.7 [-3.0-(-)1.1] | 2.5 [1.2-5.2]^ |
| Medial Heel | - |  | -1.0 [-2.3-(-)0.8] | 1.6 [0.9-2.5] |
| Lateral Heel | - |  | -1.3 [-2.7-(-)0.2] | 1.6 [1.0-2.4] |
| **Max Sensor Pressure (msp) (N/cm²)** | | | | |
| Toes 2-5 | 3.8 [1.1-8.6] | 1.2 [1.1-1.4]^ | -4.6 [-9.5-(-)1.9] | 1.5 [1.0-2.2]^ |
| Mid Foot | 3.8 [1.4-6.8] | 1.3 [1.1-1.5] | -5.8 [-8.8-(-)3.4] | 1.3 [1.1-1.6]^ |
| **Pressure Time Integral (pti) (Ns/cm²)** | | | | |
| Toes 2-5 | 0.3 [0.1-0.6] | 21.9 [4.0-120.0]^ | -0.4 [-0.8-(-)0.2] | 19.2 [1.9-199.0]^ |
| Metatarsal 1 | - |  | -0.8 [-1.2-(-)0.1] | 2.3 [0.8-7.0] |
| Metatarsal 2 | - |  | -0.7 [-1.3-(-)0.1] | 1.5 [0.8-2.8] |
| Metatarsal 3 | - |  | -0.8 [-1.5-(-)0.1) | 1.5 [0.8-2.9] |
| Metatarsal 4 | - |  | -0.7 [-1.1-(-)0.2] | 1.6 [0.8-3.3] |
| Metatarsal 5 | - |  | -0.7 [-1.1-(-)0.3] | 2.0 [0.8-5.0] |
| Mid Foot | 0.6 [0.3-1.0] | 5.1 [1.8-14.6]^ | -1.0 [-1.3-(-)-0.7] | 5.9[1.5-23.8]^ |
| Medial Heel | - |  | -0.8 [-1.4-(-)0.2] | 2.2 [1.0-5.0] |
| Lateral Heel | - |  | -0.7 [-1.4-(-)0.2] | 2.2 [1.0-5.0] |
| **Contact Area (ca) (cm²)** | | | | |
| Metatarsal 1 | -2.0 [-3.4-(-)0.4] | 1.3 [1.1-1.7]^ | -3.0 [-5.0-(-)1.6] | 1.4 [1.0-1.9]^ |
| Metatarsal 2 | - |  | -1.2 [-2.4-(-)0.3] | 1.7 [1.1-2.8]^ |
| Mid Foot | -8.4 [-13.5-(-)3.7] | 1.1 [1.0-1.2]^ | -15.1 [20.0-(-)9.7] | 1.2 [1.0-1.2]^ |
| Medial Heel | - |  | -2.2 [-3.3-(-)1.2] | 1.8 [1.1-2.9]^ |
| Lateral Heel | - |  | -1.6 [-2.5-(-)0.7] | 1.6 [1.0-2.4] |

Legend: Data displays median and [IQR] values based on the ulcerated and non-ulcerated feet of the DFU group compared to the reported maximum values in the DMC and HC groups. The Kruskal-Wallis test was used for all between group comparisons with a significance of p<0.05 and these are the main p-values reported. The Man Whitney U test was used for between two group comparisons for all significant outcomes. CI= Confidence Interval, OR= Odds Ratio, (-) = negative value. Odds Ratios were only computed from binary logistic regression analyses for variables which remained significantly different on post hoc comparisons. ^= Binary logistic regression analysis was significant at p<0.05 after adjusting for age, sex and BMI.

**Additional Table 5: Post-hoc test results and odds ratios from binary logistic regression and Cohen’s d scores**

|  | **Median difference DFU vs. DMC**  **[95% CI of difference]** | **OR**  **[95% CI]** | **Cohen’s**  **d** | **Median difference DFU vs. HC**  **[95% CI of difference]** | **OR**  **[95% CI]** | **Cohen’s**  **d** |
| --- | --- | --- | --- | --- | --- | --- |
| **Plantar Pressure (mpp)** **) (N/cm²)** | | | | | |  |
| Toes 2-5 | -0.8 [-1.5-(-)0.9] | 2.2 [1.3-3.8]^ | 0.21 | -1.0 [-1.9-(-)0.5] | 4.9 [1.3-18.0]^ | 0.40 |
| Metatarsal 1 | - |  |  | -1.5 [-2.7-(-)0.5] | 1.9 [1.1-3.4]^ | 0.36 |
| Metatarsal 5 | - |  |  | -1.1 [-2.0-(-)0.1] | 1.3 [0.9-2.0] |  |
| Mid Foot | -0.9 [-2.0-(-)0.3] | 2.0 [1.3-3.2]^ | 0.32 | -1.7 [-3.0-(-)1.1] | 2.5 [1.2-5.2]^ | 0.63 |
| Medial Heel | - |  |  | -1.0 [-2.3-(-)0.8] | 1.6 [0.9-2.5] |  |
| Lateral Heel | - |  |  | -1.3 [-2.7-(-)0.2] | 1.6 [1.0-2.4] |  |
| **Max Sensor Pressure (msp) (N/cm²)** | | | | | |  |
| Toes 2-5 | 3.8 [1.1-8.6] | 1.2 [1.1-1.4]^ | 0.29 | -4.6 [-9.5-(-)1.9] | 1.5 [1.0-2.2]^ | 0.33 |
| Mid Foot | 3.8 [1.4-6.8] | 1.3 [1.1-1.5] |  | -5.8 [-8.8-(-)3.4] | 1.3 [1.1-1.6]^ | 0.80 |
| **Pressure Time Integral (pti) (Ns/cm²)** | | | | | |  |
| Toes 2-5 | 0.3 [0.1-0.6] | 21.9 [4.0-120.0]^ | 0.45 | -0.4 [-0.8-(-)0.2] | 19.2 [1.9-199.0]^ | 0.60 |
| Metatarsal 1 | - |  |  | -0.8 [-1.2-(-)0.1] | 2.3 [0.8-7.0] |  |
| Metatarsal 2 | - |  |  | -0.7 [-1.3-(-)0.1] | 1.5 [0.8-2.8] |  |
| Metatarsal 3 | - |  |  | -0.8 [-1.5-(-)0.1) | 1.5 [0.8-2.9] |  |
| Metatarsal 4 | - |  |  | -0.7 [-1.1-(-)0.2] | 1.6 [0.8-3.3] |  |
| Metatarsal 5 | - |  |  | -0.7 [-1.1-(-)0.3] | 2.0 [0.8-5.0] |  |
| Mid Foot | 0.6 [0.3-1.0] | 5.1 [1.8-14.6]^ | 0.67 | -1.0 [-1.3-(-)-0.7] | 5.9[1.5-23.8]^ | 1.07 |
| Medial Heel | - |  |  | -0.8 [-1.4-(-)0.2] | 2.2 [1.0-5.0] |  |
| Lateral Heel | - |  |  | -0.7 [-1.4-(-)0.2] | 2.2 [1.0-5.0] |  |
| **Contact Area (ca) (cm²)** | | | | | |  |
| Metatarsal 1 | -2.0 [-3.4-(-)0.4] | 1.3 [1.1-1.7]^ | 0.44 | -3.0 [-5.0-(-)1.6] | 1.4 [1.0-1.9]^ | 0.70 |
| Metatarsal 2 | - |  |  | -1.2 [-2.4-(-)0.3] | 1.7 [1.1-2.8]^ | 0.41 |
| Mid Foot | -8.4 [-13.5-(-)3.7] | 1.1 [1.0-1.2]^ | 0.84 | -15.1 [20.0-(-)9.7] | 1.2 [1.0-1.2]^ | 1.17 |
| Medial Heel | - |  |  | -2.2 [-3.3-(-)1.2] | 1.8 [1.1-2.9]^ | 0.83 |
| Lateral Heel | - |  |  | -1.6 [-2.5-(-)0.7] | 1.6 [1.0-2.4] |  |

Legend: Data displays median and [IQR] values based on the ulcerated and non-ulcerated feet of the DFU group compared to the reported maximum values in the DMC and HC groups. The Kruskal-Wallis test was used for all between group comparisons with a significance of p<0.05 and these are the main p-values reported. The Man Whitney U test was used for between two group comparisons for all significant outcomes. CI= Confidence Interval, OR= Odds Ratio, (-) = negative value. Odds Ratios were only computed from binary logistic regression analyses for variables which remained significantly different on post hoc comparisons. ^= Binary logistic regression analysis was significant at p<0.05 after adjusting for age, sex and BMI. Cohen’s d scores were calculated for the DFU group (DFU group- control).

**Additional Table 6: Paired analysis of mean peak pressure and pressure time integral characteristics of cases**

|  | **DFU Group (n=21)** | | ***p value*** | ***Corrected p-value*** |
| --- | --- | --- | --- | --- |
|  | **Ulcerated feet (n=21)** | **Non-ulcerated feet (n=21)** |  |  |
| **Plantar Pressure (mpp)** **) (N/cm²)** | | | |  |
| Toe 1/ Hallux | 3.6 [2.5-8.2] | 5.0 [2.1-5.6] | *0.465* | *1.00* |
| Toes 2-5 | 3.0 [2.4-5.6] | 3.1 [2.1-4.5] | *0.455* | *1.00* |
| Metatarsal 1 | 5.7 [4.5-8.5] | 5.8 [4.4-7.0] | *0.232* | *1.00* |
| Metatarsal 2 | 6.8 [4.9-9.2] | 6.6 [4.4-8.0] | *0.433* | *1.00* |
| Metatarsal 3 | 6.6 [4.7-9.9] | 6.1 [4.7-6.8] | *0.191* | *1.00* |
| Metatarsal 4 | 5.7 [4.1-7.6] | 5.0 [4.0-5.8] | *0.073* | *0.73* |
| Metatarsal 5 | 4.7 [3.4-6.2] | 4.2 [2.6-5.6] | *0.126* | *1.00* |
| Mid Feet | 3.8 [3.1-6.5] | 3.2 [2.3-4.9] | *0.114* | *1.00* |
| Medial Heel | 5.8 [4.5-9.2] | 6.1 [4.0-7.1] | *0.159* | *1.00* |
| Lateral Heel | 6.3 [4.8-9.6] | 5.8 [4.0-4.6] | *0.279* | *1.00* |
| **Pressure Time Integral (pti) (Ns/cm²)** | | | |  |
| Toe 1/ Hallux | 0.9 [0.5-1.9] | 1.1 [0.7-1.6] | *0.794* | *1.00* |
| Toes 2-5 | 0.9 [0.6-1.4] | 1.0 [0.6-1.1] | *0.601* | *1.00* |
| Metatarsal 1 | 2.3 [1.5-2.9] | 2.1 [1.3-2.4] | *0.224* | *1.00* |
| Metatarsal 2 | 2.7 [1.7-3.7] | 2.3 [1.7-3.2] | *0.217* | *1.00* |
| Metatarsal 3 | 2.9 [1.7-3.9] | 2.5 [1.5-3.2] | *0.110* | *0.77* |
| Metatarsal 4 | 2.3 [2.0-3.3] | 2.1 [1.1-3.0] | *0.058* | *0.46* |
| Metatarsal 5 | 1.9 [1.3-2.7] | 1.6 [0.8-2.3] | *0.017* | *0.17* |
| Mid Feet | 1.7 [1.2-2.5] | 1.3 [0.8-1.8] | *0.030* | *0.27* |
| Medial Heel | 2.3 [1.5-3.6] | 2.3 [1.5-3.1] | *0.467* | *1.00* |
| Lateral Heel | 2.2 [1.5-3.6] | 2.3 [1.4-3.1] | *0.296* | *1.00* |

Legend: Data displays median and [IQR] values based on the ulcerated and non-ulcerated feet of the DFU group. The Wilcoxon Signed Rank test was used to compare differences between the ulcerated and non-ulcerated feet of cases. The corrected p-value indicates p-values after performing the Holm step-wise correction for multiple testing. A significance of p <0.05 was used throughout.

**Additional Table 7: Paired analysis of estimated vertical ground reaction force, contact area and maximum sensor pressure characteristics of cases**

|  | **DFU Group (n=21)** | | | ***p value*** | ***Corrected p-value*** |
| --- | --- | --- | --- | --- | --- |
|  | **Ulcerated feet (n=21)** | **Non-ulcerated feet (n=21)** | |  |  |
| **Max Sensor Pressure (msp) (N/cm²)** |  |  |  | |  |
| Toe 1/ Hallux | 17.9 [7.4-30.7] | 16.49 [7.8-24.7] | *0.414* | | *1.00* |
| Toes 2-5 | 11.7 [8.3-23.9] | 10.9 [8.5-16.0] | *0.498* | | *1.00* |
| Metatarsal 1 | 19.5 [10.8-27.5] | 12.9 [10.7-16.5] | *0.050* | | *0.05** |
| Metatarsal 2 | 18.0 [12.7-21.4] | 16.2 [11.3-21.2] | *0.639* | | *1.00* |
| Metatarsal 3 | 17.0 [12.3-24.3] | 14.1 [9.9-21.3] | *0.205* | | *1.00* |
| Metatarsal 4 | 14.1 [10.6-15.9] | 10.8 [7.4-16.1] | *0.205* | | *1.00* |
| Metatarsal 5 | 11.7 [8.1-15.8] | 8.4 [5.5-13.8] | *0.068* | | *0.54* |
| Mid Feet | 12.4 [9.4-19.4] | 9.9 [6.3-13.8] | *0.058* | | *0.52* |
| Medial Heel | 17.1 [11.9-26.7] | 15.6 [8.7-22.1] | *0.159* | | *1.00* |
| Lateral Heel | 16.2 [11.4-28.2] | 15.2 [8.8-19.5] | *0.181* | | *1.00* |
| **Contact Area (ca) ( cm²)** |  |  |  | |  |
| Toe 1/ Hallux | 11.0 [8.4-13.3] | 11.0 [8.3-13.2] | | *0.205* | *1.00* |
| Toes 2-5 | 10.1 [8.0-11.7] | 7.4 [6.0-11.6] | | *0.099* | *0.89* |
| Metatarsal 1 | 12.8 [10.2-15.2] | 9.4 [7.4-14.1] | | *0.144* | *1.00* |
| Metatarsal 2 | 10.0 [8.7-11.9] | 9.3 [7.8-11.4] | | *0.187* | *1.00* |
| Metatarsal 3 | 8.9 [7.5-9.7] | 8.3 [6.5-9.6] | | *0.106* | *0.89* |
| Metatarsal 4 | 9.5 [8.5-10.3] | 9.1 [6.8-10.4] | | *0.076* | *0.76* |
| Metatarsal 5 | 8.5 [6.6-10.6] | 8.6 [7.4-10.2] | | *0.986* | *1.00* |
| Mid Feet | 37.4 [28.1-42.4] | 30.1 [25.5-38.0] | | *0.232* | *1.00* |
| Medial Heel | 17.4 [15.5-18.8] | 16.3 [13.3-18.7] | | *0.375* | *1.00* |
| Lateral Heel | 14.9 [13.8-15.7] | 14.3 [10.8-17.2] | | *0.330* | *1.00* |

Legend: Data displays median and [IQR] values based on the ulcerated and non-ulcerated feet of the DFU group. The Wilcoxon Signed Rank test was used to compare differences between the ulcerated and non-ulcerated feet of cases. The corrected p-value indicates p-values after performing the Holm step-wise correction for multiple testing. A significance of p <0.05 was used throughout.
